# Therapeutic Effects of Risperidone against Spinal Cord Injury in a Rat Model of Asphyxial Cardiac Arrest: A Focus on Body Temperature, Paraplegia, Motor Neuron Damage, and Neuroinflammation

**DOI:** 10.3390/vetsci8100230

**Published:** 2021-10-13

**Authors:** Tae-Kyeong Lee, Jae-Chul Lee, Hyun-Jin Tae, Hyung-Il Kim, Myoung Cheol Shin, Ji Hyeon Ahn, Joon Ha Park, Dae Won Kim, Seongkweon Hong, Soo Young Choi, Jun Hwi Cho, Moo-Ho Won

**Affiliations:** 1 Department of Biomedical Science, Research Institute for Bioscience and Biotechnology, Hallym University, Chuncheon 24252, Gangwon, Korea; tk_lee@hallym.ac.kr (T.-K.L.); sychoi@hallym.ac.kr (S.Y.C.); 2 Department of Neurobiology, School of Medicine, Kangwon National University, Chuncheon 24341, Gangwon, Korea; anajclee@kangwon.ac.kr (J.-C.L.); jh-ahn@ysu.ac.kr (J.H.A.); 3 Bio-Safety Research Institute, College of Veterinary Medicine, Chonbuk National University, Iksan 54596, Jeollabuk, Korea; hjtae@jbnu.ac.kr; 4 Department of Emergency Medicine, Dankook University Hospital, College of Medicine, Dankook University, Cheonan 31116, Chungnam, Korea; hilovesjj@naver.com; 5 Department of Emergency Medicine, Kangwon National University Hospital, School of Medicine, Kangwon National University, Chuncheon 24289, Gangwon, Korea; dr10126@naver.com; 6 Department of Physical Therapy, College of Health Science, Youngsan University, Yangsan 50510, Gyeongnam, Korea; 7 Department of Anatomy, College of Korean Medicine, Dongguk University, Gyeongju 38066, Gyeongbuk, Korea; jh-park@dongguk.ac.kr; 8 Department of Biochemistry and Molecular Biology, Research Institute of Oral Sciences, College of Dentistry, Gangnung-Wonju National University, Gangneung 25457, Gangwon, Korea; kimdw@gwnu.ac.kr; 9 Department of Surgery, Kangwon National University Hospital, School of Medicine, Kangwon National University, Chuncheon 24289, Gangwon, Korea; skhong1@kangwon.ac.kr

**Keywords:** whole-body ischemia, cardiopulmonary resuscitation, drug-induced hypothermia, spinal motor neuron, inflammation, paraplegia

## Abstract

Cardiac arrest (CA) causes severe spinal cord injury and evokes spinal cord disorders including paraplegia. It has been reported that risperidone, an antipsychotic drug, effectively protects neuronal cell death from transient ischemia injury in gerbil brains. However, until now, studies on the effects of risperidone on spinal cord injury after asphyxial CA (ACA) and cardiopulmonary resuscitation (CPR) are not sufficient. Therefore, this study investigated the effect of risperidone on hind limb motor deficits and neuronal damage/death in the lumbar part of the spinal cord following ACA in rats. Mortality, severe motor deficits in the hind limbs, and the damage/death (loss) of motor neurons located in the anterior horn were observed two days after ACA/CPR. These symptoms were significantly alleviated by risperidone (an atypical antipsychotic) treatment after ACA. In vehicle-treated rats, the immunoreactivities of tumor necrosis factor-alpha (TNF-α) and interleukin 1-beta (IL-1β), as pro-inflammatory cytokines, were increased, and the immunoreactivities of IL-4 and IL-13, as anti-inflammatory cytokines, were reduced with time after ACA/CPR. In contrast, in risperidone-treated rats, the immunoreactivity of the pro-inflammatory cytokines was significantly decreased, and the anti-inflammatory cytokines were enhanced compared to vehicle-treated rats. In brief, risperidone treatment after ACA/CPR in rats significantly improved the survival rate and attenuated paralysis, the damage/death (loss) of motor neurons, and inflammation in the lumbar anterior horn. Thus, risperidone might be a therapeutic agent for paraplegia by attenuation of the damage/death (loss) of spinal motor neurons and neuroinflammation after ACA/CPR.

## 1. Introduction

CA abruptly ceases blood circulation and oxygen delivery to the entire body, induces ischemia in the whole body, and develops high mortality after CA/CPR [1,2]. Studies on CA have concentrated on the improvement in the rate of the return of spontaneous circulation (ROSC) after CPR [3,4]. It has been reported that CA is one of the causes of severe spinal cord injuries including paraplegia, which negatively affects the quality of life in patients [5,6,7]. It is well known that motor neurons located in the ventral horn of the spinal cord are very vulnerable to ischemia-reperfusion injury [8,9,10]. However, the factors protecting or attenuating the damage of spinal motor neurons following ischemic insults have been insufficiently reported yet.

It is well accepted that body temperature influences the outcome of ischemic injury in patients after the ROSC [11,12,13,14]. To date, hypothermia has been applied to increase the ROSC in order to improve the survival rate of patients with CA. Data using experimental animals indicate that early cooling after the ROSC provides neurological recovery, but delayed hypothermia after ROSC limits these beneficial effects [15,16].

Risperidone (RIS), a benzoxazole derivative, has been widely used as a second-generation antipsychotic drug and selective monoaminergic antagonist containing high affinity for serotonin type 2 (5-HT_2A_) and dopamine type 2 (D_2_) receptors in the limbic system [17,18]. Studies in 2003 and 2004 reported that RIS induced hypothermia in patients with brain disorders, such as schizophrenia [19,20]. In a recent experimental study, RIS induced hypothermia in gerbils and effectively protected cells or neurons from ischemia-reperfusion injury in the hippocampus by attenuating glial activation and maintaining antioxidants [21].

Neuroinflammation is a major pathophysiologic feature following brain ischemic insults [22,23]. The inflammatory cascade is induced a few hours after ischemic insults, and inflammation may last for a few days or weeks as a delayed tissue reaction to the damage [24,25]. The inflammatory response is controlled through the balance between pro- and anti-inflammatory cytokines, and this balance disappears after ischemia [26]. It is well accepted that pro-inflammatory cytokines promote inflammatory processes and the processes worsen following ischemia-reperfusion, but anti-inflammatory cytokines inhibit pro-inflammatory cytokine expression and induce ischemic tolerance [27,28].

There are some explanations of the protective effects of hypothermia against ischemic damage in the spinal cord [29,30], and we hypothesized that treatment with RIS after asphyxial CA (ACA) attenuates paraplegia and affects neuroinflammation in the spinal cord of patients with ACA. In this regard, we developed a rat model of ACA and examined the effects of RIS on paraplegia, neuronal damage and death, and inflammatory cytokines in the lumbar part of the spinal cord in rats following ACA/CPR.

## 2. Materials and Methods

### 2.1. Rats, Protocol, and Groups for Experiment

Male Sprague-Dawley rats at 10 weeks of age (body weight, 310–320 g) were obtained from the Experimental Animal Center of Kangwon National University (Chuncheon, Republic of Korea). The rats were kept under pathogen-free conditions with suitable temperature (about 23 °C) and humidity (about 60%). Freely accessible feed (DBL Co., Ltd.; Chungbuk, Korea) and water were provided to the rats. A 12-h cycle of light and dark was maintained.

The protocol for this experiment was approved on 18 February 2020 (approval no., KW-200113-1) by the Institutional Animal Care and Use Committee (IACUC). The protocol content adhered to the guidelines, which are in compliance with the “Current International Laws and Policies” from the “Guide for the Care and Use of Laboratory Animals” (The National Academies Press, 8th Ed., 2011) [31]. The number of the rats used in this study was minimized, and the suffering caused by the procedures used in this experiment was minimized.

**Figure 1:**
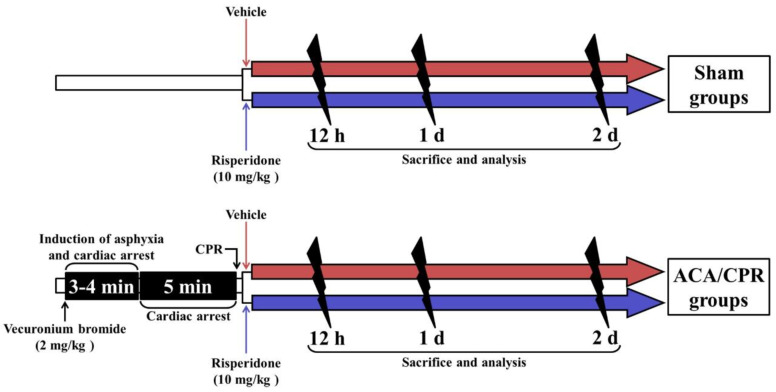
Experimental timeline. The rats used in this study underwent sham or ACA/CPR followed by treatment with vehicle or 10 mg/kg RIS. They were deeply anesthetized and sacrificed at 12 h, 1 day, and 2 days after ROSC, and their spinal cords were used for analyses.

Rats (total *n* = 84) were assigned to four groups and treated as follows (Figure 1): (1) Sham+vehicle group (*n* = 21), which was given identical anesthetic and sham ACA/CPR operation, and intraperitoneally injected with vehicle; (2) ACA/CPR+vehicle group (*n* = 21 at each point in time), which was given ACA/CPR operation and intraperitoneally injected with vehicle; (3) Sham+RIS group (*n* = 21 at each point in time), which was given sham ACA/CPR operation and intraperitoneally injected with RIS; and (4) ACA/CPR+RIS group (*n* = 21), which was given ACA/CPR operation and injected intraperitoneally with RIS. In each group, seven rats were sacrificed at 12 h, one day and two days after ACA/CPR.

For reference, the original number of the rats used in this study was different (*n* = used number/original number) due to the survival rate as follows: (1) Sham+vehicle group (*n* = 7/7 at each time); (2) ACA/CPR+vehicle group (*n* = 7/9 at 12 h; *n* = 7/16 one day; *n* = 7/164 two days); (3) Sham+RIS group (*n* = 7/7 at each time); (4) ACA/CPR+RIS group (*n* = 7/8 at 12 h; *n* = 7/8 one day; *n* = 7/11 two days).

### 2.2. ACA/CPR Operation and RIS Treatment

As shown in Figure 1, ACA/CPR was performed. Each rat was anesthetized with 2.5% isoflurane (Hana Pharmaceutical Co., Ltd.; Seoul, Korea) (in 33% oxygen and 67% nitrous oxide) and endotracheally intubated with a cannula (14-gauge) under mechanical ventilation with 2% isoflurane (in 33% oxygen and 67% nitrous oxide). Under the anesthesia, the right femoral artery and vein were isolated and cannulated with catheters (PE-50) to administer drug and to monitor arterial blood pressure. During the surgery of ACA/PCR, the body temperature in the rats was monitored using a rectal temperature probe (TR-100) (Fine Science Tools, Foster City, CA, USA) and maintained at a normothermic condition (37 ± 0.5 °C) using a thermometric blanket (Harvard Apparatus™, Holliston, MA, USA). Two mg/kg of vecuronium bromide obtained from Reyon Pharmaceutical (Seoul, Korea) was intravenously injected at 5 min after stabilization, and the anesthesia was stopped. Then, the mechanical ventilation in the rats was stopped, and the endotracheal tube was removed from the ventilator. Usually, ACA was confirmed at 3–4 min after vecuronium bromide injection in this study. Perfect ACA was confirmed when pulseless electric activity (PEA) was shown and mean arterial pressure (MAP) was below 25 mmHg [8,9]. ACA was maintained for 5 min. Then, CPR was immediately initiated by an intravenous injection of 0.005 mg/kg of epinephrine (Dai Han Pharm, Seoul, Korea) and 1 meq/kg of sodium bicarbonate (Daewon Pharm, Seoul, Korea), and mechanical ventilation with 100% oxygen was simultaneously given. Subsequently, manual chest compressions were performed. Namely, manual chest compression was performed at a rate of 300/min until MAP increased to 60 mmHg, and electrocardiography was checked [8,9]. Once each rat breathed and was hemodynamically stable, which was usually shown 1 h after ROSC, the catheter was removed. The rat came out from the anesthesia 1 h after ROSC. For the control of body temperature from 20 min to 6 h after ACA, any artificial maintenance for body temperature was not conducted after ROSC while the ambient temperature (room temperature) was kept at 24 ± 1 °C.

In this study, the rats of the sham group underwent the surgical procedure of ACA without the injection of vecuronium. After the surgical procedure, the rats were placed in cages (DBL Co., Ltd.; Chungbuk, Korea), in which aspen beds were spread on the bottom, and they were kept in thermal incubators (Mirae Medical Industry, Seoul, Korea) at 25 °C and 60% humidity. While the rats were kept in the incubators, room temperature was maintained at 24 ± 1 °C. Body temperature and MAP was recorded every minute from 0 to 20 min. Thereafter, till 1 h after ACA induction, body temperature and MAP was measured every 5 min. Especially, body temperature was recorded every 15 min from 1 to 6 h after ACA induction.

As shown in Figure 1, vehicle or RIS (10 mg/kg) (Sigma-Aldrich, St. Louis, MO, USA) was injected into the peritoneal cavity immediately after ACA/CPR operation. The dose of RIS was selected based on a previous study [21]. RIS was dissolved in 0.3% Tween 80 (in 0.85% saline; NaCl *w*/*v*; Junsei Chemical Co., Ltd., Tokyo, Japan).

### 2.3. Assessment of Physiological Variables and Motor Function

Body weight and MAP between the groups were compared at 1 day after ROSC. Motor function of the hind limbs was evaluated for paralysis at 1 day after ROSC using Tarlov Scale [8]: motor deficit scoring 0, complete paralysis with no hind limb function; 1, slight movement in articulations; 2, unable to stand without support; 3, sit alone; 4, weak walking with poor jumping; 5, normal walking.

### 2.4. Preparation of Histological Sections

The rats (*n* = 7 at each point in time) in each group were used for histopathological staining and immunohistochemistry at 12 h, 1 day, and 2 days after ROSC. The rats were deeply anesthetized by intraperitoneal injection of 200 mg/kg pentobarbital sodium (JW pharm Co Ltd., Seoul, Korea) [32]. Under the anesthesia, they were transcardially rinsed with 0.1 M phosphate-buffered saline (PBS, pH 7.4) and fixed with 4% paraformaldehyde (in 0.1 M PB, pH 7.4) for 30 min. The lumbar parts of the spinal cords were obtained and postfixed in the same fixative for 8 h. The lumbar spinal cords were infiltrated with 25% sucrose (in 0.1 M PB) to be cryoprotected for 12 h. To prepare histological sections, the spinal cord tissues were frozen in a cryostat (Leica, Wetzlar, Germany) and serially cut into a 25-µm coronal plane.

### 2.5. Fluoro-Jade B (F-J B) Histofluorescence

F-J B (a fluorescent marker for cellular degeneration) histofluorescence was performed to assess neuronal damage/death (loss) after ACA/CPR. In short, as described previously [33], the spinal cord sections were immersed in 0.0004% F-J B (Histochem, Jefferson, AR, USA) and washed. Finally, for the reaction of the F-J B, these sections were placed on a slide warmer (about 50 °C).

To quantitatively analyze the death or protection of motor neurons in the ventral horn, five sections were chosen with a 120-μm interval. F-J B-positive cells were counted as previously described [34]. In short, F-J B-positive cells (neurons) were observed with an epifluorescence microscope (BX53) (Olympus, Tokyo, Japan) with blue (450–490 nm) excitation light. The images were captured with a digital camera (DP7) (Olympus, Tokyo, Japan) connected to a PC monitor. The F-J B-positive cells were counted in 200,000 μm^2^ (400 μm × 500 μm) at the anterior horn. Counts of the cells were evaluated by averaging the total numbers obtained from 35 sections from 7 rats/group using an image analyzing system (Optimas 6.5) from CyberMetrics (Scottsdale, AZ, USA).

### 2.6. Immunohistochemistry

In this study, general immunohistochemistry was carried out to examine changes regarding the neurons, pro-inflammatory, and anti-inflammatory cytokines. For the immunohistochemistry, we used primary antibodies as follows: mouse anti-neuronal nuclei (NeuN; diluted 1:1100; Cat. No., MAB377; Chemicon International, Temecula, CA, USA), rabbit anti-TNF-α (diluted 1:1200) (Cat. No., ab66579; Abcam, Cambridge, UK), rabbit anti-IL-1β (diluted 1:250) (Cat. No., ab2105; Abcam, Cambridge, UK), goat anti-IL-4 (diluted 1:200) (Cat. No. sc-1260; Santa Cruz Biotechnology, Santa Cruz, CA, USA), and goat anti-IL-13 (diluted 1:200) (Cat. No., sc-393365; Santa Cruz Biotechnology, Santa Cruz, CA, USA). In short, as described previously [35], the sections were incubated with each diluted antibody for 12 h at 4 °C. After the sections were washed, they were reacted with biotinylated horse anti-mouse (diluted 1:200) (Cat. No., BA-2001;Vector Laboratories, Burlingame, CA, USA), goat rabbit (diluted 1:200) (Cat. No., BA-1000; Vector Laboratories, Burlingame, CA, USA), or rabbit anti-goat IgG (diluted 1:200) (Cat. No., BA-5000; Vector Laboratories, Burlingame, CA, USA) and, thereafter, developed by avidin-biotin complex (ABC) (diluted 1:300) (Cat. No. PK-4000; Vector Laboratories, Burlingame, CA, USA). Finally, they were visualized with 3,3′-diaminobenzidine solution (DAB; Cat. No., D8001; Sigma-Aldrich, St. Louis, MO, USA). The sections were identically reacted with DAB solution for 90 s at room temperature. In addition, negative control tests for NeuN, TNF-α, IL-1 β, IL-4, and IL-13 were performed for the specificity of each immunostaining, with pre-immune serum instead of each primary antibody. As a result, any immunostained structures were not shown in the tested sections.

For quantitative analysis of the number of NeuN immunoreactive motor neurons and their change, five sections/rat were chosen with a 120-μm interval. The numbers were counted as described in the Section 2.5.

For quantitative analysis of each immunoreactivity (TNF-α, IL-1 β, IL-4, and IL-13) in the ventral horn, the images were taken using the above-mentioned method and analyzed as described in our published paper [35]. Briefly, each image of the captured immunoreactivity was evaluated as optical density (OD): the OD was obtained after transforming each immunoreactive structure to mean gray level using the formula OD = log (256/mean gray level). Finally, each OD was compared as the relative optical density (ROD): a ratio of the ROD was evaluated as percent using Image J software (version 1.59) from NIH (Bethesda, MD, USA).

### 2.7. Statistical Analysis

In this study, SPSS software (version 15.0) from SPSS Inc (Chicago, IL, USA) was used to perform all statistical analysis. We used the Kolmogorov and Smirnov test for testing normal distributions and Bartlett test for testing the identical standard error of the means (SEMs), and all our data passed the normality test. The statistical significances of the mean among the experimental groups were determined by one-way analysis of variance followed by post hoc Tukey test for all pairwise multiple comparisons. Any differences lower than 0.05 of *p* value were considered significant.

## 3. Results

### 3.1. Changes in Physiological Function and Body Temperature

**Figure 2:**
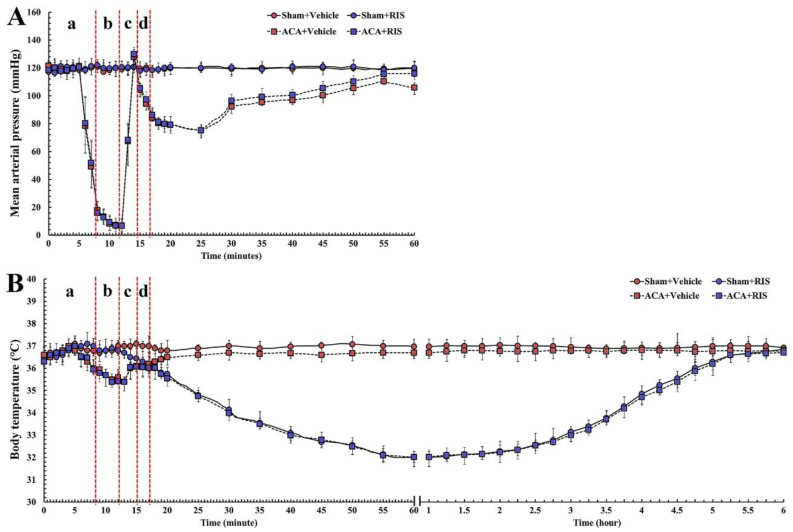
MAP (**A**) and body temperature (**B**) before, during, and after ACA in the Sham+vehicle, ACA/CPR+vehicle, and ACA/CPR+RIS groups. Note that body temperature in the ACA/CPR+RIS group was 33 ± 0.5 °C from 1 to 2 h after ACA. a, inducing ACA; b, maintaining ACA condition; c, conducting CPR; d, confirming ROSC. The bars indicate the means ± SEM (*n* = 7).

MAP and body temperature was recorded in each group before and after ACA operation as shown in Figure 2. Before ACA, MAP and body temperature were similar to the baselines observed in the Sham+vehicle group. Body temperature in the ACA/CPR+RIS group was not significantly different from that in the ACA/CPR+vehicle group (Figure 2A). Under 24 ± 1 °C of room temperature, a significant low body (rectal) temperature (33 ± 0.5 °C) in all RIS groups was detected from 1 to 2 h after ACA, which was due to RIS injection. Thereafter, their body temperature was spontaneously and gradually increased with intermittently shivering to 37 ± 0.5 °C (Figure 2B).

### 3.2. Survival Rate and Motor Deficit Score

**Figure 3:**
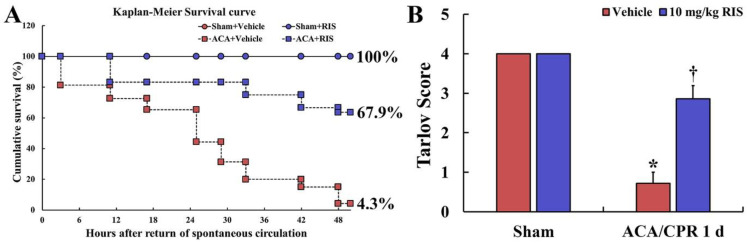
Kaplan-Meier survival curve and Tarlov score (**A**) Survival rate (*p* < 0.05) in the Sham+vehicle, Sham+IRS, ACA/CPR+vehicle, and ACA/CPR+RIS groups using Kaplan–Meier analysis for 2 days after ACA/CPR. The ACA/CPR+RIS group reveals a higher survival rate than the ACA/CPR+vehicle group. At 2 days after ACA/CPR, the cumulative survival rate in the ACA/CPR+vehicle group is 4.3% whereas the cumulate survival rate in the ACA/CPR+RIP group is 67.9%. (**B**) Motor function of both hind limbs in the Sham+vehicle, Sham+RIS, ACA/CPR+vehicle, and ACA/CPR+RIS groups using Tarlov Scoring System. At 1 day after ACA, a significant higher score in the ACA/CPR+RIP group is observed compared to that in the ACA/CPR+vehicle group. The bars indicate the means ± SEM (*n* = 7; * *p* < 0.05 vs. Sham+vehicle group; ^†^ *p* < 0.05 vs. ACA/CPR+vehicle group).

The survival rate in the ACA/CPR+vehicle and ACA/CPR+vehicle groups was recorded by Kaplan-Meier analysis for 2 days after ACA/CPR (Figure 3A). In all sham groups, all rats survived (Figure 3A). In the ACA/CPR+vehicle group, the survival rate gradually reduced with time after ACA/CPR, showing 65.3% at 1 day and 4.3% at 2 days after ROSC (Figure 3A). In the ACA/CPR+RIS group, however, the survival rate was significantly high compared with that in the ACA/CPR+vehicle group, showing 92.4% at 1 day and 67.9% at 2 days after ACA/CPR (Figure 3A).

Hind limb motor deficit (paralysis) was evaluated with the Tarlov score at 1 day after ACA/CPR (Figure 3B). The rats of the Sham+vehicle group revealed normal function in their hind limbs. In the ACA/CPR+vehicle group, the score was significantly low (average 0.8 point) compared with that in the Sham+vehicle group (average 4.1 point) (*p* < 0.01). In the ACA/CPR+RIS group, however, motor function was significantly better (average 2.9 point) than that in the ACA/CPR+vehicle group (*p* < 0.05).

### 3.3. Neuroprotection by RIS

#### 3.3.1. NeuN Immunoreactive Neurons

**Figure 4:**
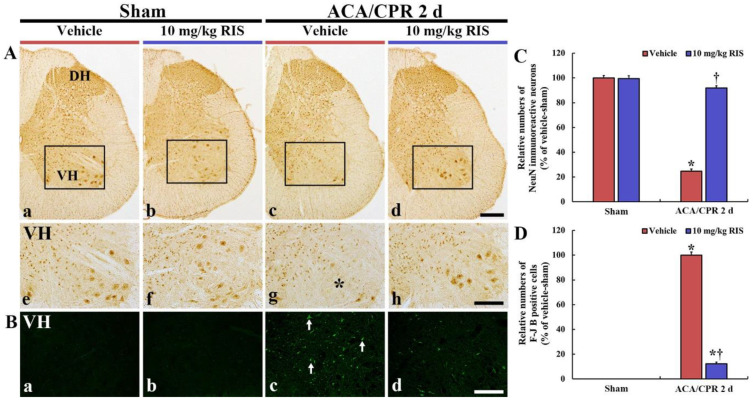
NeuN immunohistochemistry and F-J B histofluorescence (**A**) NeuN immunohistochemistry in the lumbar spinal cord of the Sham+vehicle (**a**,**e**), Sham+RIS (**b**,**f**), ACA/CPR+vehicle (**c**,**g**), and ACA/CPR+RIS (**d**,**h**) groups at 2 days after ACA/CPR. The middle panels are high magnified images for the squares in the upper panels. In the ACA/CPR+vehicle group, NeuN+ neurons are rarely shown (asterisk) in the ventral horn (VH). However, many NeuN+ cells are shown in the ACA/CPR+RIS group. DH, dorsal horn. Scale bar = 200 (**a**–**d**) and 100 (**e**–**h**) µm. (**B**) F-J B histofluorescence in the ventral horn of the Sham+vehicle (**a**), Sham+RIS (**b**), ACA/CPR+vehicle (**c**), and ACA/CPR+RIS (**d**) groups at 2 days after ACA/CPR. In the ACA/CPR+vehicle group, many F-J B+ cells (arrows) are shown, but the numbers of F-J B+ cells are decreased in the ACA/CPR+RIS group. Scale bar = 400 µm (**A**) and 100 µm. (**C**,**D**) Quantitative analyses of NeuN+ (**C**) and F-J B+ cells (**D**) in the VH. The bars indicate the means ± SEM (*n* = 7; * *p* < 0.05 vs. Sham+vehicle group; ^†^ *p* < 0.05 vs. ACA/CPR+vehicle group).

We examined neuronal damage/loss in the ventral horn of the lumbar part in the spinal cord after ACA/CPR using immunohistochemistry with NeuN: NeuN is well used to detect neuronal nucleus damage (Figure 4). In the Sham+vehicle and Sham+RIS groups, neurons in the anterior horn, which are called motor neurons, were well stained with NeuN in their nuclei (Figure 4A(a,b,e,f)). In the ACA/CPR+vehicle group, a few neurons stained with NeuN (NeuN^+^ neurons) were shown in the anterior horn at 2 days after ACA/CPR (Figure 4A(c,g)). The mean percentage of NeuN^+^ neurons, in this group, was 24.6% of that in the Sham+vehicle group (Figure 4C). However, in the ACA/CPR+RIS group, many NeuN^+^ neurons were found at 2 days after ACA/CPR (Figure 4A(d,h)), revealing that the mean percentage of the motor neurons was 91.7% of that in the Sham+vehicle group (Figure 4C).

#### 3.3.2. F-J B-Positive Cells

The neuroprotection by RIS from ACA/CPR in the ventral horn was analyzed by F-J B histofluorescence: F-J B is an excellent marker for detection of dead cells (neurons) (Figure 4B). No F-J B-positive (F-J B^+^) cells were found in the Sham+vehicle and Sham+RIS groups (Figure 4B(a,b)). In the ACA/CPR+vehicle group, many F-J B^+^ cells were found in the anterior horn at 2 days after ACA/CPR (Figure 4Bc,D). In the ACA/CPR+RIS group, the numbers of F-J B^+^ cells were significantly decreased at 2 days after ACA/CPR (Figure 4Bd), showing that the mean percentage of the F-J B^+^ cells was 12.1% of that in the ACA/CPR+vehicle group (Figure 4D).

### 3.4. Decreased Pro-Inflammatory Cytokines by RIS

#### 3.4.1. TNF-α Immunoreactivity

**Figure 5:**
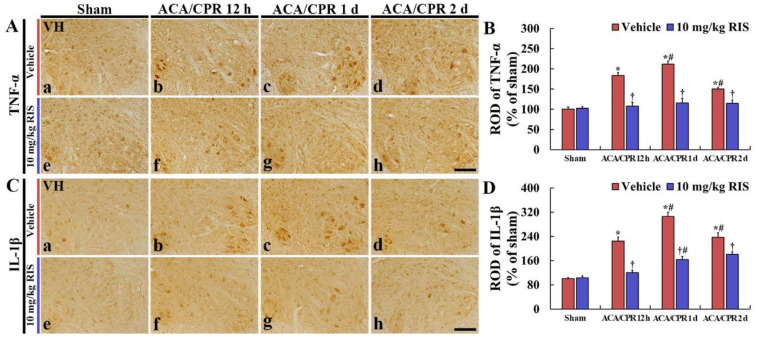
Immunohistochemical staining for TNF-α and IL-1β (**A**,**C**) Immunohistochemistry for TNF-α (**A**) and IL-1β (**C**) in the ventral horn of the Sham+vehicle (**a**), ACA/CPR+vehicle (**b**–**d**), Sham+RIS (**e**), and ACA/CPR+RIS (**f**–**h**) groups at 12 h, 1 day, and 2 days after ACA/CPR. In the ACA/CPR+vehicle group, TNF-α and IL-1β immunoreactivities are significantly increased from 12 h after ACA/CPR. However, in the ACA/CPR+RIS group, immunoreactivities of TNF-α and IL-1β are significantly low compared with that shown in the ACA/CPR+vehicle group. VH, ventral horn. Scale bar = 100 µm. (**A**,**C**) RODs of TNF-α (**B**) and IL-1β (**D**) immunoreactivity. The bars indicate the means ± SEM (*n* = 7; * *p* < 0.05 vs. Sham+vehicle group; ^†^ *p* < 0.05 vs. ACA/CPR+vehicle group; ^#^ *p* < 0.05 vs. Pre-time point of corresponding group).

TNF-α immunoreactivity shown in the Sham+vehicle group was shown in the motor neurons located in the anterior horn (Figure 5Aa). In the ACA/CPR+vehicle group, TNF-α immunoreactivity was gradually enhanced until 1 day after ACA, showing that the ROD of TNF-α immunoreactivity at 12 h and 1 day after ACA/CPR was 183.5% and 211.9%, respectively, compared with that in the Sham+vehicle group (Figure 5A(b,c),B). Thereafter, TNF-α immunoreactivity was decreased, but the ROD was 150.1% of that in the Sham+vehicle group (Figure 5Ad,B).

In the Sham+RIS group, TNF-α immunoreactivity in the ventral horn was similar to that in the Sham+vehicle group (Figure 5Ae,B). In addition, in the ACA/CPR+RIS group, TNF-α immunoreactivity in the anterior horn showed no difference from that in the Sham+vehicle group (Figure 5A(f–h),B).

#### 3.4.2. IL-1 β Immunoreactivity

In the Sham+vehicle group, IL-1β immunoreactivity was weakly shown in the motor neurons (Figure 5Ca). In the ACA/CPR+vehicle group, IL-1β immunoreactivity at 12 h, 1 day, and 2 days after ACA/CPR was intensely increased, showing that the ROD was 224.4%, 305.3, and 237.1%, respectively, compared with that in the Sham+vehicle group (Figure 5B(b–d),C).

In the Sham+RIS group, IL-1β immunoreactivity in the lumbar ventral horn was not significantly different from that found in the Sham+vehicle group (Figure 5Be,D). In the ACA/CPR+RIS group, IL-1β immunoreactivity was gradually enhanced after ACA, but the ROD at each point in time was significantly lower (41.2%, 45.4%, and 23.5%, respectively) than that in the ACA/CPR+vehicle group (Figure 5D).

### 3.5. Increased Anti-Inflammatory Cytokines by RIS

#### 3.5.1. IL-4 Immunoreactivity

**Figure 6:**
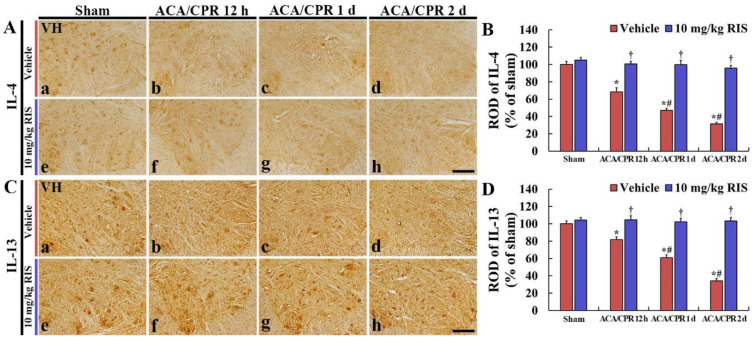
Immunohistochemical staining for IL-4 and IL-13 (**A**,**C**) Immunohistochemistry for IL-4 (**A**) and IL-13 (**C**) in the ventral horn of the Sham+vehicle (**a**), ACA/CPR+vehicle (**b**–**d**), Sham+RIS (**e**), and ACA/CPR+RIS (**f**–**h**) groups at 12 h, 1 day, and 2 days after ACA/CPR. In the ACA/CPR+vehicle group, immunoreactivities of IL-4 and IL-13 are significantly decreased from 12 h after ACA/CPR. However, in the ACA/CPR+RIS group, immunoreactivities of IL-4 and IL-13 are maintained after ACA/CPR. VH, ventral horn. Scale bar = 100 µm. (**A**,**C**) RODs of IL-4 (**B**) and IL-13 (**D**) immunoreactivity. The bars indicate the means ± SEM (*n* = 7; * *p* < 0.05 vs. Sham+vehicle group; ^†^ *p* < 0.05 vs. ACA/CPR+vehicle group; ^#^ *p* < 0.05 vs. Pre-time point of the corresponding group).

IL-4 immunoreactivity in the ventral horn of the Sham+vehicle group was shown in the motor neurons (Figure 6Aa). In the ACA/CPR+vehicle group, IL-4 immunoreactivity was dramatically and gradually decreased after ACA/CPR, showing that the ROD at 12 h, 1 day, and 2 days after ACA/CPR was 68.3%, 47.1%, and 31.3%, respectively, compared with that found in the Sham+vehicle group (Figure 6A(b–d),B).

In the Sham+RIS group, IL-4 immunoreactivity in the lumbar ventral horn was similar to that shown in the Sham+vehicle group (Figure 6Ae,B). In the ACA/CPR+RIS group, IL-4 immunoreactivity in the anterior horn was maintained after ACA/CPR (Figure 6A(f–h),B).

#### 3.5.2. IL-13 Immunoreactivity

In the ventral horn of the Sham+vehicle group, IL-13 immunoreactivity was also found in the motor neurons (Figure 6Ba). IL-13 immunoreactivity in the ACA/CPR+vehicle group was dramatically and gradually decreased after ACA/CPR (RODs: 81.7% at 12 h, 60.9% at 1 day, and 34.5% at 2 days after ACA/CPR) compared with that in the Sham+vehicle group (Figure 6B(b–d),D).

In the Sham+RIS group, IL-13 immunoreactivity in the ventral horn was not different from that shown in the Sham+vehicle group (Figure 6Ce,D). In the ACA/CPR+RIS group, IL-13 immunoreactivity in the anterior horn was also maintained after ACA/CPR (Figure 6C(f–h),D).

## 4. Discussion

Generally, the spinal cord is prominently susceptible to ischemic insults owing to diverse circulatory abnormalities [36,37,38]. In addition, the vulnerability and sensitivity of the spinal cord after ACA/CPR are completely different from those of the brain. In particular, the time course of cell death in the ischemic spinal cord is different from that in the ischemic brain after ACA/CPR [39]. This difference may be because much energy is required for the extensive activity of the motor neurons located in the anterior horn of the spinal cord [36]. It has been reported that thoracic aortic occlusion-induced spinal cord ischemia leads to neuronal damage in the ventral horn of the lumbar spinal cord from one day after spinal cord ischemia in rats [40]. In a rat model of ACA/CPR, neuronal death (loss) in the anterior horn in the lumbar part of the spinal cord occurs at one day after ACA/CPR [9]. In addition, Ahn et al. [10] recently reported that neuronal death in the central nervous system (CNS) autonomic control center (myelencephalon and thoracolumbar division of the spinal cord) occurred very early compared to the other CNS divisions after ACA/CPR in rats. In our current study, we found that ventral motor neurons at the level of the lumbar spinal cord were dead at two days after ACA/CPR in rats. These results indicated that the time course of neuronal damage/death in the CNS following global ischemia in the whole body (i.e., ACA) must be different according to the regions of the brain and spinal cord and that the spinal cord has a higher vulnerability to transient ischemia than the brain. In short, spinal cord damage following ACA/CPR occurred much faster than brain damage.

Hind-limb paralysis is one of the main disorders after ACA/CPR [8,9]. Duggal and Lach [39] reported that selective vulnerability of the lumbosacral part of the spinal cord was shown in patients with ACA/CPR and hypotension. Experimental studies on ischemic spinal cord injury have been conducted using animal models with aortic disease or local vascular change [40,41,42]. In a rabbit model of spinal cord ischemia, which is simply produced by occlusion of the spinal arteries that have no collateral circulation, paraplegia occurs when motor neurons in the lumbar spinal cord are damaged or dead after ischemic-reperfusion injury [43], and the death of motor neurons in the lumbar spinal cord is shown within one day after ischemia-reperfusion [44,45,46]. In our current study using a rat model of ACA/CPR, paralysis in the hind limbs was seen one day after ACA/CPR, and most motor neurons located in the anterior horn were not seen two days after ACA/CPR. Taken together, we suggest that paraplegia following ACA/CPR might occur with motor neuron damage or death because normal motor nerve fibers (general somatic efferent) via the spinal nerves cannot innervate muscles of the limbs [47].

For several decades, RIS, as a selective monoaminergic antagonist, has been widely used for the treatment of schizophrenia [17,18]. In addition, RIS has been reported to induce hypothermia [19,21,48]. It has been reported that hypothermia can display neuroprotection and improve damaged outcomes in experimental animal models of spinal cord and brain injury [49]. However, few data concerning the effects of hypothermia against spinal cord injury after ACA have been accumulated. In this regard, we examined the effect of RIS on motor deficits in the hind limbs and its related neuronal vulnerability in the spinal cord following ACA/CPR in rats. It was reported that RIS treatment after brain transient ischemia induced hypothermia within 30 min and lasted for four hours and that hypothermia displayed effective protection against the death of hippocampal neurons induced by transient brain ischemia by attenuating glial activation and maintaining antioxidant enzymes [21]. In our present study, the effects of RIS-induced hypothermia on spinal cord injury after ACA/CPR in rats were investigated, and, as expected, RIS-induced hypothermia significantly improved paraplegia and alleviated the damage/death (loss) of ventral motor neurons at two days after ACA/CPR. These results strongly suggest that RIS treatment after ACA improves neurological dysfunction by attenuating the damage of the ventral motor neurons in patients with spinal cord injury from ACA.

Over the past few years, a body of evidence has stressed the roles of inflammation in the pathophysiology of acute brain ischemia [50]. Cytokines include many groups of inflammatory mediators, and they act as signaling molecules to control inflammation and to induce positive or negative effects on neuronal survival [51]. It is well known that pro-inflammatory cytokines are involved in the amplification of inflammatory reactions and contribute to the pathogenesis of neurological disorders, whereas anti-inflammatory cytokines are decisively involved in resolving inflammation through downregulating the production of pro-inflammatory cytokines [52].

Some studies showed the anti-inflammatory properties of RIS in an in vivo and in vitro model. MacDowell K.S. et al. [53] demonstrated the anti-inflammatory effect of RIS. In detail, a single administration of RIS regulated various factors that triggered advanced inflammatory responses, such as the expression of inflammatory cytokines (interleukin (IL)-1β and tumor necrosis factor (TNF)-α) following lipopolysaccharide (LPS)-induced inflammation in the frontal cortex of rat brains. Additionally, a precedent study showed that RIS suppressed the production of pro-inflammatory cytokines and decreased the level of inducible NO synthase (iNOS), which are secreted by reactive microglia using a microglial cell line [54]. In this study, the immunoreactivity of pro-inflammatory cytokines (TNF-α and IL-1β) in the ventral horn of the ACA/CPR+vehicle group was increased with time after ACA/CPR, but, in the ACA/CPR+RIS group, the immunoreactivity of TNF-α and IL-1β was significantly lower than that in the ACA/CPR+vehicle group. It has been found that TNF-α and IL-1β are activated in the brains of animal models of transient brain ischemia as mediators in response to ischemic injury [55,56,57]. TNF-α and IL-1β play critical roles in post-ischemic inflammatory injury in the spinal cord [58,59,60]. Hasturk et al. [58] concluded that serum TNF-α and IL-1β levels significantly increased after spinal cord ischemia-reperfusion injury accompanied by tissue damage. In rat models of spinal cord ischemia-reperfusion injury, increased levels of cytokines induced by ischemic injury were observed to be associated with the deterioration of motor function and histological damage in the spinal cord [61] and TNF-*α* levels were significantly increased within 1.5 h, and peaked 3 h after ischemic injury [62]. In a swine model of spinal cord ischemia-reperfusion injury, TNF-*α* levels were significantly increased from 6 to 24 h after ischemic injury [63]. Additionally, IL-1 expression was significantly increased in the spinal cord 6 and 36 h following ischemic-reperfusion injury in mice [64]. Taken together, we suggest that pro-inflammatory cytokines might contribute to cell death in the spinal cord following ischemia-reperfusion injury. In addition, our current findings indicate that RIS treatment after ACA/CPT induces hypothermia and prevents the abnormal expressions of TNF-α and IL-1β in the ischemic spinal cord.

In our current study, significant decreases in the immunoreactivity of anti-inflammatory cytokines (IL-4 and IL-13) were observed in the anterior horn cord in the lumbar spinal cord after ACA/CPR. However, IL-4 and IL-13 immunoreactivity in the ACA/CPR+RIS group was not reduced compared to that in the sham+vehicle group. It was demonstrated that the sustained or increased expression of endogenous anti-inflammatory cytokines (IL-4 and IL-13) contributed to neuronal survival from ischemia-reperfusion injury in the gerbil hippocampus after transient forebrain ischemia [65,66]. Additionally, some studies showed that IL-4 and IL-13 suppressed the expression and production of pro-inflammatory cytokines (TNF-α and IL-1β) in the spinal cord of animal models of spinal cord ischemia [67,68]. Therefore, taken together, the findings suggested that the maintained expression of anti-inflammatory cytokines in the ACA/CPR+RIS group may contribute to the protection of motor neurons from ACA injury.

## 5. Conclusions

In brief, our present findings showed that RIS treatment after ACA/CPR induced hypothermic conditions, significantly reduced mortality, and attenuated hind-limb paralysis. In addition, neuronal damage/death (loss) in the ventral horn of the lumbar spinal cord was ameliorated. These might be associated with the significant decreases of pro-inflammatory cytokines and the maintenance of anti-inflammatory cytokines, which might be induced by the hypothermic condition induced by RIS treatment. Taken together, we suggest that immediate post-treatment with RIS after ACA can be utilized as a novel therapeutic approach of patients with ACA.

## Data Availability

The data presented in this study are available on request from the corresponding author.

## Abbreviations

ACA	asphyxial cardiac arrest
CNS	central nervous system
CPR	cardiopulmonary resuscitation
F-J B	fluoro-Jade B
IL	interleukin
NeuN	risperidone
RIS	major histocompatibility complex
ROSC	return of spontaneous circulation
ROD	relative optical density
TNF-α	tumor necrosis factor α
VH	ventral horn
